# Transcriptomics-based identification of shared biomarkers across type 2 diabetes, mild cognitive impairment, and uric acid metabolism

**DOI:** 10.3389/ebm.2026.11060

**Published:** 2026-07-06

**Authors:** Yan Liu, Dongmei Kang, Yuan Lei

**Affiliations:** 1 Endocrinology Department, The First Affiliated Hospital of Anhui Medical University, Hefei, Anhui, China; 2 Anhui Public Health Clinical Center, Hefei, Anhui, China; 3 International Medical Department, The First Affiliated Hospital of USTC, Anhui Provincial Hospital, Hefei, Anhui, China

**Keywords:** biomarkers, immune infiltration, mild cognitive impairment, type 2 diabetes mellitus, uric acid metabolism

## Abstract

Uric acid metabolism is associated with the development of type 2 diabetes mellitus (T2DM), cardiometabolic, and cardiovascular diseases. Additionally, T2DM patients often exhibit mild cognitive impairment (MCI). However, the underlying mechanisms remain unclear. This study aims to identify and validate biomarkers associated with uric acid metabolism in T2DM and MCI, with the goal of discovering potential diagnostic and therapeutic targets to improve the quality of life for T2DM patients. Transcriptomic data for T2DM, MCI and uric acid metabolism-related genes were sourced from public databases. Biomarkers were screened using machine learning and validated for expression. Subsequent analyses included functional enrichment, immune infiltration, subcellular localization, and drug prediction. Three biomarkers—HP, ITGB3, and SELP—were identified. All showed significantly elevated expression in the T2DM group (p < 0.05). HP and ITGB3 were primarily enriched in ribosome-related pathways, primary immunodeficiency, and adherens junction processes. Immune infiltration analysis revealed that immature B cells and plasmacytoid dendritic cells were significantly enriched in T2DM. HP showed the strongest positive correlation with plasmacytoid dendritic cells (cor = 0.65, FDR <0.05), while ITGB3 exhibited the strongest positive correlation with immature B cells (cor = 0.76, FDR <0.05). Several potential therapeutic drugs were predicted, including calcifediol (score = −99.93) and meclofenamic acid (score = −99.89). This study identified three candidate biomarkers co-dysregulated across T2DM and MCI transcriptomes and associated with uric acid metabolism. Given the exploratory sample sizes, these findings are considered hypothesis-generating and require validation in larger independent cohorts.

## Impact statement

Using transcriptomic data from public databases (GSE26168, GSE95849, GSE18309) combined with LASSO and XGBoost machine learning, this study identified three candidate biomarkers—HP, ITGB3, and SELP—co-dysregulated across T2DM, MCI, and uric acid metabolism gene sets. These genes are implicated in inflammatory signalling, platelet activation, and vascular pathways based on functional enrichment and immune infiltration analyses. All three achieved AUC >0.9 in an independent validation cohort (n = 12) and showed consistent upregulation by RT-qPCR (n = 5 per group), providing hypothesis-generating evidence for their potential utility in early identification of T2DM patients at risk of cognitive decline. CMap-based drug prediction nominated calcifediol and meclofenamic acid as candidates warranting experimental investigation. Findings are exploratory and require validation in larger independent cohorts before clinical inference can be drawn.

## Introduction

Type 2 diabetes mellitus (T2DM) is a common metabolic disorder characterized by hyperglycemia, insulin resistance, and relative insulin deficiency [1]. Since the 1970s, the overall prevalence of T2DM in China has continued to rise, increasing rapidly with age [2]. T2DM not only affects metabolic homeostasis but also directly damages the central nervous system, leading to cognitive decline and a heightened risk of dementia [3]. Studies indicate that 25–36% of diabetic patients exhibit mild cognitive impairment (MCI), and T2DM can accelerate the progression from MCI to dementia [4, 5]. MCI represents a transitional state between normal aging and dementia and is recognized as a cognitive impairment syndrome [6]. In China, the number of individuals with MCI is estimated between 38 and 77 million, with incidence rates on the rise [7]. Insulin resistance plays a pivotal role in the pathogenesis of both T2DM and MCI, contributing to impaired glucose metabolism, increased oxidative stress, and persistent hyperinsulinemia and hyperglycemia [6]. Brain insulin resistance appears to be an early and common feature in Alzheimer’s disease (AD) patients, and dysregulated glucose metabolism may be a dominant factor in dementia progression, though the precise mechanisms remain elusive [8]. While previous studies have established an association between T2DM and MCI, the specific interactive mechanisms require further elucidation.

Notably, insulin resistance and abnormal uric acid metabolism are mutually reinforcing. Insulin resistance can induce hyperuricemia by impairing renal uric acid transport [9], while hyperuricemia exacerbates insulin resistance through oxidative stress and inflammation, creating a vicious cycle [10]. Uric acid metabolism involves the synthesis, breakdown, and excretion of purine-derived uric acid in the body [11]. Hyperuricemia is an independent risk factor for diabetes, chronic kidney disease, cardiovascular disease, and stroke [12]. Urate crystals can stimulate vascular walls, promote atherosclerosis, damage pancreatic β-cells, induce insulin resistance, and further disrupt glucose metabolism [13, 14].

Additionally, both low and high serum uric acid levels have been linked to cognitive decline in MCI patients [15, 16]. The complex interrelationships among T2DM, MCI, and abnormal uric acid metabolism underscore the need to identify relevant biomarkers. In-depth exploration of such biomarkers could facilitate early diagnosis, improve cognitive prognosis in T2DM patients, and delay dementia progression.

This study aims to systematically screen and validate biomarkers related to MCI and uric acid metabolism in T2DM using transcriptomics and experimental approaches. We employed bioinformatics to analyze biological pathways, immune microenvironment associations, and potential therapeutic drugs, offering new insights for clinical intervention.

## Materials and methods

### Data collection

Data were obtained from the Gene Expression Omnibus (GEO) database.^1^ The GSE26168 dataset (platform GPL6883) served as the training set, comprising 9 T2DM whole blood samples and 8 healthy controls (7 impaired fasting glucose samples were excluded) [17]. The GSE95849 dataset (platform GPL22448) was used as the validation set, including 6 T2DM peripheral blood samples and 6 healthy controls (6 diabetic peripheral neuropathy samples were excluded) [18]. The GSE18309 dataset (platform GPL570) served as the MCI training set, containing 3 MCI whole blood samples and 3 healthy controls (3 AD samples were excluded) [19]. All datasets were downloaded from GEO as preprocessed Series Matrix files (background-corrected and normalized). Data were checked for distribution and log2-transformed if needed; probes with missing values were removed, and the probe with the highest mean expression was retained per gene. The analyses were performed separately for each dataset without cross-platform merging, and integration was conducted at the gene-symbol level by intersection, avoiding batch effects.

Additionally, 3,632 uric acid metabolism-related genes were retrieved from the Genecards database^2^ using the keyword “Uric Acid” [20] (Supplementary Table 1). To assess specificity, a biologically constrained sensitivity analysis was performed by intersecting the GeneCards gene set with KEGG purine metabolism genes and GO terms related to uric acid metabolism (e.g., inflammatory response, platelet activation, and cell adhesion), yielding 585 refined genes (Supplementary Table 1). The broad GeneCards-derived gene set was used for primary analysis to maximize sensitivity, while the constrained gene set served as a confirmatory sensitivity analysis.

### Identification of differentially expressed genes (DEGs)

Differential expression analysis was performed on the T2DM training set (GSE26168) using the limma package (v3.54.0) [21]. T2DM-DEGs were defined with thresholds of |log_2_Fold Change (FC)| > 1 and p < 0.05. Volcano plots were generated with ggplot2 (v3.4.1) [22], annotating the top 10 upregulated and downregulated genes. Heatmaps were constructed using ComplexHeatmap (v2.14.0) [23] and circlize package (v0.4.15) [24] to visualize expression patterns.

Simultaneously, to obtain the DEGs between MCI and control groups in the training set GSE18309, this study performed differential analysis using the limma package (v3.54.0) on MCI and control samples. MCI-DEGs were defined with thresholds of |log_2_Fold Change (FC)| > 0.5 and p < 0.05 to ensure statistical and biological significance. The ggplot2 package (v3.4.1) was used to generate a volcano plot, in which the top 10 most significantly up-regulated and top 10 most significantly down-regulated genes (sorted by p value from low to high) were annotated. Additionally, a heatmap was constructed with the ComplexHeatmap package (v2.14.0) to visualize the expression patterns of the top 10 up-regulated and top 10 down-regulated genes.

### Identification and functional enrichment of candidate genes

In order to obtain candidate genes associated with T2DM, MCI, and uric acid metabolism, the VennDiagram package (v1.7.1) [25] was used to intersect the sets of T2DM-DEGs, MCI-DEGs, and uric acid metabolism-related genes, and this study defined the intersection genes as candidate genes. As a sensitivity analysis, the same intersection procedure was repeated using the 585 biologically constrained uric acid-related genes to evaluate the robustness of candidate gene identification against gene set definition breadth.

Subsequently, Gene Ontology (GO) and Kyoto Encyclopedia of Genes and Genomes (KEGG) analyses were conducted to determine the potential biological functions and mechanisms associated with the candidate genes, employing the clusterProfiler package (v4.2.2) [26] (p < 0.05). The ggplot2 package (v3.4.1) was used to visualize the top 10 GO enrichment terms sorted by p value from low to high. Among them, GO comprised 3 functional dimensions, namely, molecular function (MF), cellular component [27], and biological process (BP). KEGG pathway enrichment revealed the classic immune pathways and cellular signaling mechanisms in which these candidate genes were involved.

Given the small candidate gene set (n = 11), the protein-protein interaction (PPI) network was constructed using the Search Tool for the Retrieval of Interacting Genes/Proteins (STRING) online database (;^3^ interaction score >0.15), not for hub screening, but to provide a protein-level interaction context supporting the functional coherence of the genes prior to machine learning selection. The Cytoscape package (v3.8.2) [28] was used to visualize the PPI network results. Robustness was assessed via sensitivity analysis at a medium confidence threshold (score >0.4), followed by GeneMANIA^4^ co-expression networking to validate the functional hub status of the candidate genes.

### Machine learning and expression validation

In order to further identify candidate biomarkers associated with T2DM and MCI, this study performed the least absolute shrinkage and selection operator (LASSO) and extreme gradient boosting (XGBoost) algorithm on the GSE26168. Specifically, the glmnet package (v4.1-8) [29] was used to perform LASSO regression analysis. The variables were determined at minimum lambda (10-fold cross-validation), and genes with non-zero regression coefficients were selected as LASSO candidate characteristic genes. To maximize data utility and test feature selection robustness for our small sample (n = 17), we also used leave-one-out cross-validation (LOOCV) to assess the robustness of feature selection against different data partitioning strategies. Given the small sample size (n = 17, p = 11), standard logistic regression was not applied due to complete separation and unstable coefficient estimates observed in preliminary analyses. Instead, LASSO was preferred as it effectively reduces overfitting through L1 regularization.

Meanwhile, the xgboost package (v2.0.3.1) [30] was employed to extract the core metric Gain, which measured feature importance. And the top-ranked genes based on Gain were selected as the XGBoost candidate characteristic genes. In addition, the ggvenn package (v0.1.9) [31] was used to identify the intersection of the candidate characteristic genes from 2 algorithms and defined these overlapping genes as the characteristic genes. For methodological robustness, univariate logistic regression was conducted as a sensitivity analysis.

To evaluate the ability of characteristic genes to distinguish T2DM and control samples and avoid circularity, area under curve (AUC)-based validation of the identified characteristic genes was performed with pROC package (v1.18.0) [32] in the independent external dataset GSE95849 (n = 12), which was not used in the feature selection step. The training set GSE26168 AUC values are also reported for reference only. Subsequently, characteristic genes that exhibited AUC >0.7 in both the training and validation sets were selected as candidate biomarkers. Gene expression analysis was subsequently conducted on these candidate biomarkers by comparing T2DM and control samples from the GSE26168 and GSE95849 datasets. Based on the candidate biomarkers, the Wilcoxon test was used to compare the expression differences of the candidate biomarkers between the 2 groups (p < 0.05), and box plots were drawn using the ggplot2 package (v3.4.1). Candidate biomarkers that showed significant differences between the 2 groups and consistent expression trends were selected as biomarkers.

### Gene set enrichment analysis (GSEA)

To further investigate the relevant signaling pathways and biological information associated with biomarkers in T2DM, this study downloaded the reference gene set (c2. cp.kegg.v7.4. symbols.gmt) from the molecular signatures database (MsigDB,^5^). Based on biomarkers and all other genes in the dataset, the Spearman correlation coefficient was calculated using the psych package (v2.1.6) [33]. The genes were then sorted from largest to smallest using the correlation coefficient as the sorting criterion. Subsequently, GSEA was performed employing the clusterProfiler package (v4.2.2), with 1,000 permutations and thresholds of |Normalised enrichment score (NES)| > 1, p < 0.05. The same GSEA procedure was additionally applied to the MCI dataset (GSE18309) using HP and ITGB3 as core genes, to explore pathway enrichment patterns in the MCI context and compare them with T2DM findings.

### Immune infiltration analysis and correlation analysis among biomarkers

To assess differences in immune cell infiltration between the T2DM and control samples, the GSVA package (v1.50.0) [34] was used to quantify the relative abundance of 28 immune cell types across all samples in the training set GSE26168. The infiltration abundance of immune cells was then visualized using the pheatmap package (v1.0.12) [35]. Moreover, the Wilcoxon test was conducted to identify immune cells exhibiting notable disparities between T2DM and control groups, with p-values adjusted using the Benjamini-Hochberg (BH) method to control the false discovery rate (FDR); adjusted p < 0.05 was considered significant. The ggplot2 package (v3.4.1) was used for box plot visualization. To analyze the correlations between differential immune cells, Spearman correlation analysis was performed using the psych package (v2.1.6). Furthermore, to further investigate the relationship between biomarker expression and immune cell infiltration, the psych package (v2.1.6) was employed to calculate Spearman correlation coefficient between biomarkers and the immune cell enrichment scores. Analyses were conducted with the thresholds of |correlation coefficient(cor)| > 0.3 and BH-adjusted p < 0.05.

To gain deeper insights into the correlations among biomarkers, Spearman correlation analysis was performed using the psych package (v2.1.6) based on the T2DM and control samples from the training set GSE26168. Correlations were identified with p < 0.05 and |cor| > 0.3. To assess immune microenvironment changes in MCI, the same immune infiltration analysis pipeline was independently applied to the GSE18309 dataset, with BH-FDR correction applied to all comparisons.

### Tissue and cellular localization analysis

To investigate the distribution of biomarkers across tissues, the Human Proteome Atlas (HPA,^6^) database was used to explore the expression levels of biomarkers in different tissues. Visualization of biomarker expression levels across tissues was achieved using histograms. The top 5 tissue distribution data for each biomarker were imported into Cytoscape software (v3.8.2) to generate tissue-biomarker network diagrams.

To further identify the specific subcellular localization of the biomarkers, this study used the Genecards^2^ database to obtain the subcellular distribution information of the biomarkers. The list of subcellular localization data with confidence = 5 was selected.

### Computational prediction of candidate compounds and traditional Chinese medicines

First, to explore potential therapeutic drugs, this study conducted Connectivity Map (CMap) analysis to predict small-molecule compounds for T2DM. Specifically, drug features were extracted from the Connectivity Map database.^7^ Biomarkers served as input data. The final analysis results were assigned a score ranging from −100 to 100. Negative scores indicated the ability to reverse gene expression, signifying potential therapeutic significance. CMap profiles are mainly derived from cancer cell lines; thus, drug predictions are purely computational and hypothesis-generating, requiring validation in disease-relevant models before therapeutic conclusions can be made. Simultaneously, the biomarkers were imported into the Coremine Medical database,^8^ where relevant Traditional Chinese Medicine (TCM) information pertaining to the biomarkers was downloaded. Potential therapeutic traditional Chinese medicines were identified at a significance level of p < 0.05.

### Reverse transcription quantitative polymerase chain reaction (RT-qPCR)

The expression levels of these biomarkers were verified through the application of RT-qPCR. Samples of blood were collected from patients diagnosed with T2DM and healthy control subjects at Anhui Public Health Clinical Center,The First Affiliated Hospital of Anhui Medical University, North District, with each sample representing an independent biological replicate (n = 5 per group). Prior to sample collection, informed consent was duly obtained from each participant, and the study protocol had been approved by the Ethics Committee of Anhui Public Health Clinical Center(Approval No. PJ-YX2025-044). Detailed clinical characteristics of all participants, including age, sex, BMI, HbA1c, and current medications, are presented in Supplementary Table 12.

For the extraction of total RNA, the TRIzol (R401-01) reagent (produced by Novozyme Biotech Co., Ltd., located in Nanjing, China) was employed. The extraction process was meticulously conducted in strict accordance with the manufacturer - provided protocol. Subsequently, the reverse transcription of the extracted total RNA into cDNA was executed using the HP All-in-one qRT Master Mix II RT203-Ver.1 for qPCR, which was manufactured by Kunming Yungeng Biotechnology Co., Ltd. The reverse transcription procedure was carried out precisely as per the instructions provided by the supplier. The RT-qPCR reactions were performed with the utilization of the ® 2×Universal Blue SYBR Green qPCR Master Mix (supplied by Servicebio, based in Wuhan, China). The primer sequences utilized for the polymerase chain reaction amplification were comprehensively detailed in Supplementary Table 2. To exclude gDNA contamination, cDNA synthesis was performed using a kit with integrated gDNA digestion (Yeasen, Cat. No. 11141ES60). Amplification curves displayed typical sigmoidal kinetics; despite the absence of formal efficiency calculation, specificity was supported by sigmoidal profiles and single-peak melt curves, indicating no non-specific amplification or primer-dimer formation. Although SELP primers were initially designed against a predicted transcript (XM accession), NCBI Primer-BLAST confirmed the amplicon resides within a conserved coding region identical to the curated reference (NM_003005), ensuring valid mRNA-level detection. Among various candidate genes, GAPDH was specifically selected as the internal reference gene for normalization purposes. Each sample was assayed in technical triplicate, and the mean Ct value was used for subsequent calculations. The relative expression levels of the biomarkers were calculated by implementing the 2^−ΔΔCT^ method, as previously described and validated in a published study [36]. After the completion of data acquisition from the RT-qPCR experiments, the resultant data were initially exported to Microsoft Excel for preliminary organization. Subsequently, the data underwent in - depth analysis and visualization using GraphPad Prism 10.1.2 software^9^ (p < 0.05), which is a widely recognized and highly utilized tool in the field of scientific data analysis and graphical representation.

### Serum quantification of haptoglobin, integrin β1, and P-selectin by ELISA

Serum concentrations of HP, ITGβ1, and SELP in T2DM patients (n = 11) and healthy controls (n = 10) were measured using commercially available enzyme-linked immunosorbent assay (ELISA) kits (Fenxibio, Beijing, China): HP (Cat# FXs202379), ITGβ1 (Cat# FXs207165), and SELP (Cat# FXs205897). All assays were performed in accordance with the manufacturer’s instructions.

### Statistical analysis

All bioinformatic analyses were performed in the R language (v4.2.2). Data from different cohorts were compared using the Wilcoxon test. The Spearman method was utilized for correlation analysis. Differences were regarded as significant at p < 0.05. Post hoc power analysis was performed based on qPCR expression data using a two-sample t-test framework (two-sided, α = 0.05). Effect sizes (Cohen’s d) were calculated from the mean and standard deviation of each group.

## Results

### The 11 candidate genes were linked to T2DM, MCI, and uric acid metabolism

**FIGURE 1:**
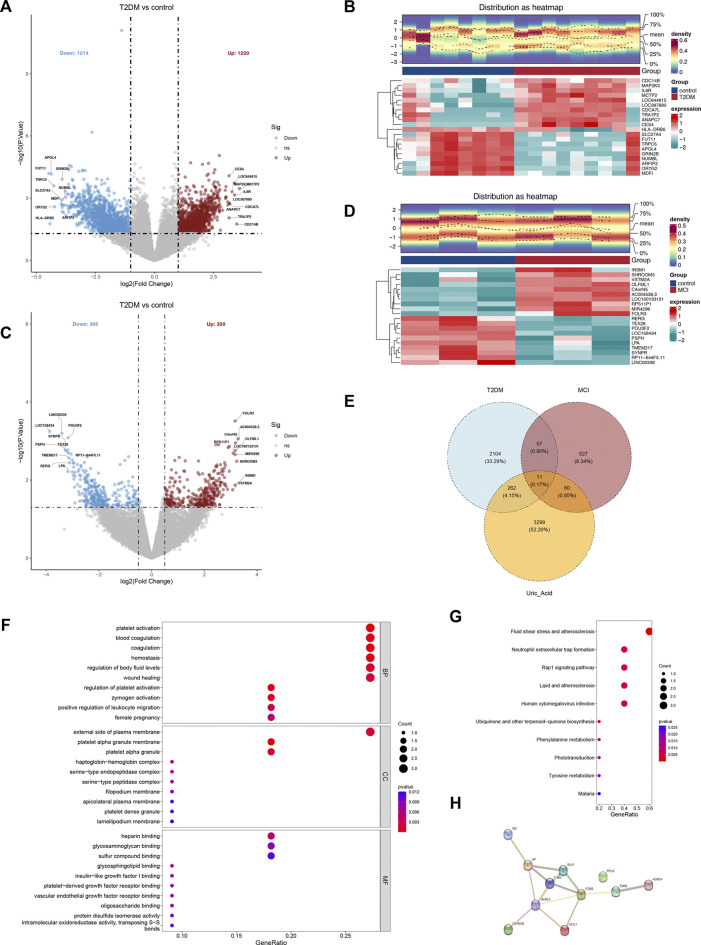
Identification and functional exploration of candidate genes linking T2DM, MCI, and uric acid metabolism. **(A)** Volcano plot of differentially expressed genes (DEGs) between T2DM patients (n = 9) and healthy controls (n = 8) in the GSE26168 dataset. Red dots indicate significantly upregulated genes, blue dots indicate significantly downregulated genes, and gray dots represent non-significant genes. The top 10 upregulated and downregulated genes are annotated. **(B)** Heatmap showing expression patterns of the top 10 upregulated and top 10 downregulated T2DM-DEGs across samples. Each row represents a gene, each column represents a sample, and color intensity indicates expression level. **(C)** Volcano plot of DEGs between MCI patients (n = 3) and healthy controls (n = 3) in the GSE18309 dataset. Red dots indicate significantly upregulated genes, blue dots indicate significantly downregulated genes. **(D)** Heatmap showing expression patterns of the top 10 upregulated and top 10 downregulated MCI-DEGs across samples. **(E)** Venn diagram showing the intersection of T2DM-DEGs, MCI-DEGs, and uric acid metabolism-related genes. **(F)** Gene Ontology (GO) enrichment analysis of the 11 candidate genes, showing the top 10 enriched terms across Biological Process (BP), Cellular Component [27], and Molecular Function (MF). Bubble size represents gene count, color represents adjusted P-value. **(G)** Kyoto Encyclopedia of Genes and Genomes (KEGG) pathway enrichment analysis of the 11 candidate genes, showing significantly enriched pathways (P < 0.05). **(H)** Protein-protein interaction (PPI) network of the candidate genes. Nodes represent proteins, edges represent interactions.

Principal component analysis (PCA) confirmed acceptable within-dataset sample distributions across all three datasets, with no outlier samples detected (Supplementary Figure 1). Differential expression analysis identified 2,434 T2DM-DEGs, with 1,220 upregulated and 1,214 downregulated genes in T2DM versus controls. The top differentially expressed genes included APOL4, GRIN2B, and TRPC5 (downregulated), as well as CES4 and IL6R (upregulated) (Figure 1A). The heatmap similarly demonstrated expression patterns of these genes in T2DM and control groups (Figure 1B), indicating differential activation or suppression in T2DM. Similarly, a total of 655 MCI-DEGs was identified between MCI and control groups. Compared to the control samples, there were 350 up-regulated genes (such as FOLR3, OLFML1, and RPS11P1) and 305 down-regulated genes (such as LINC00339 and LOC158434) in the MCI samples (Figures 1C,D). Finally, a total of 11 intersection genes between the 2,434 T2DM-DEGs, 655 MCI-DEGs, and 3,632 uric acid metabolism-related genes were identified as candidate genes (Figure 1E). As a sensitivity analysis using the biologically constrained 585-gene set, intersection with T2DM-DEGs and MCI-DEGs yielded five genes (FSTL1, HP, ITGB3, SELP, and THBD), all overlapping with the original 11 candidates (Supplementary Figure 2A).

The functional enrichment analysis of these candidate genes revealed associations with 177 GO terms (p < 0.05). Among these, the BPs mainly involved the processes of platelet activation and blood coagulation. The CCs primarily included the components of platelet alpha granule membrane, external side of plasma membrane, and haptoglobin-hemoglobin complex. In terms of MFs, the candidate genes were mainly enriched in the functions of heparin binding and organic acid binding (Figure 1F; Supplementary Table 3). Additionally, 18 KEGG pathways were identified, including Fluid shear stress and atherosclerosis, Neutrophil extracellular trap formation, and Rap1 signaling pathway (p < 0.05) (Figure 1G; Supplementary Table 4). These results suggested that the candidate genes might be involved in immune system, signal transduction and metabolism of cofactors and vitamins. The PPI network (11 nodes, 14 edges) provided protein-level interaction context (Figure 1H); sensitivity analysis at a higher threshold (>0.4) and GeneMANIA co-expression analysis consistently identified HP, SELP, ITGB3, THBD, and FSTL1 as functionally coherent hubs (Supplementary Figures 2B,2C), supporting the biological relevance of the candidate gene set. The results showed these genes might influence each other directly or indirectly, and might participate in the biological processes linking the uric acid metabolism to T2DM and MCI.

### The HP, ITGB3, and SELP were identified as biomarkers

**FIGURE 2:**
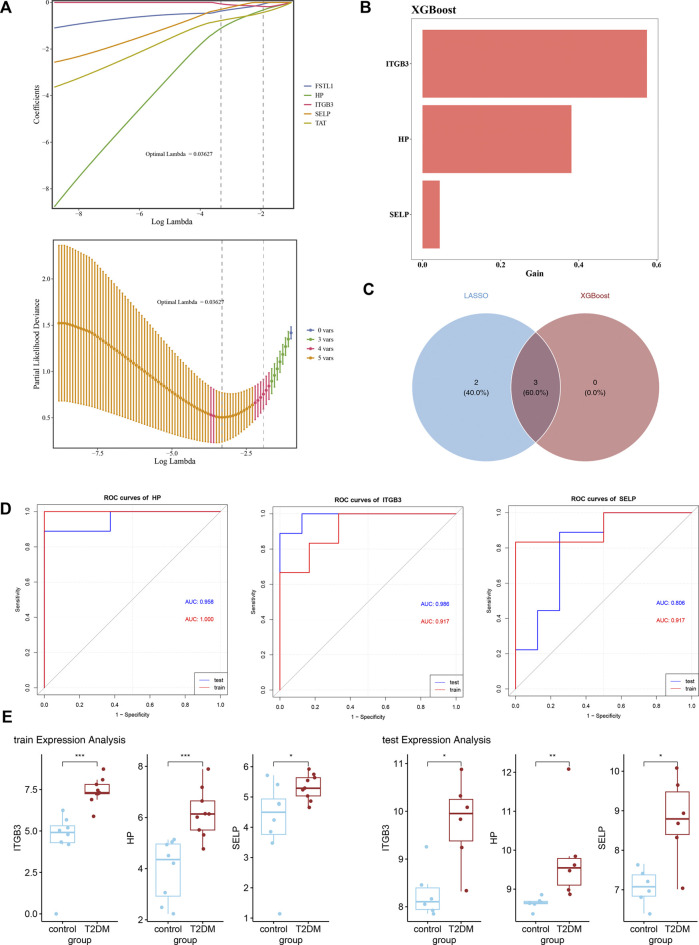
Screening and validation of biomarkers using machine learning algorithms. **(A)** LASSO regression cross-validation curve for feature selection from the 11 candidate genes using the GSE26168 dataset. **(B)** XGBoost feature importance ranking based on Gain metric. **(C)** Venn diagram showing the intersection of LASSO-selected genes and XGBoost-selected genes. **(D)** Receiver operating characteristic [37] curves evaluating the diagnostic performance of HP, ITGB3, and SELP in distinguishing T2DM patients from healthy controls. Upper panel shows results from the training set (GSE26168, T2DM n = 9, control n = 8). Lower panel shows results from the validation set (GSE95849, T2DM n = 6, control n = 6). Area under the curve (AUC) values are displayed in each panel **(E)** Box plots comparing expression levels of HP, ITGB3, and SELP between T2DM patients and healthy controls. Left panel shows results from the training set (GSE26168). Right panel shows results from the validation set (GSE95849). The horizontal line inside each box represents the median, box boundaries indicate the interquartile range, and whiskers extend to 1.5 times the interquartile range. P-values were calculated using the Wilcoxon rank-sum test. *P < 0.05, **P < 0.01, ***P < 0.001 indicate statistically significant differences.

In the LASSO regression analysis, the optimal value of lambda. min = 0.03627 and 5 LASSO candidate characteristic genes including HP, ITGB3, TAT, FSTL1, and SELP were ultimately identified (Figure 2A). Notably, the LOOCV feature selection results were identical to those obtained from the original 10-fold cross-validation (Supplementary Table 5). Through the XGBoost model and Gain-based ranking, 3 XGBoost candidate characteristic genes (HP, ITGB3, and SELP) were selected from the candidate genes (Figure 2B). Subsequently, this study intersected the genes obtained from the mentioned above machine learning algorithms and finally found that 3 genes (HP, ITGB3, and SELP) could be indicated by all the algorithms, meaning that these genes could be used as characteristic genes (Figure 2C). As a sensitivity analysis, univariate logistic regression across 11 candidate genes (Supplementary Table 6) indicated that HP, ITGB3, and SELP showed the largest effect sizes. Stepwise logistic regression selected HP and TAT, but complete separation resulted in unstable estimates. These results provide supportive evidence for the robustness of the LASSO/XGBoost-based feature selection.

To avoid circularity, biomarker performance was primarily evaluated in the independent GSE95849 dataset. HP (AUC = 1.000, 95% CI: 1.000–1.000), ITGB3 (AUC = 0.917, 95% CI: 0.759–1.000), and SELP (AUC = 0.917, 95% CI: 0.738–1.000) all demonstrated strong discriminatory ability (AUC >0.7). However, given the small sample size, the result AUC = 1.0 should be interpreted with caution as it may reflect potential overestimation of performance. The results observed in the training dataset (AUC: 0.806–0.986) are provided for reference only and showed a consistent trend (Figure 2D). Accordingly, these 3 characteristic genes were identified as candidate biomarkers. Next, the expression of HP, ITGB3, and SELP showed significant differences in both 2 datasets, and all of them showed a significant upward trend in the T2DM group (Figure 2E). Finally, these 3 candidate biomarkers were identified as biomarkers. The results reinforced the correlation between biomarkers and disease states, as well as their potential biological significance.

### Strongly correlated biomarkers were significantly enriched in multiple signaling pathways

**FIGURE 3:**
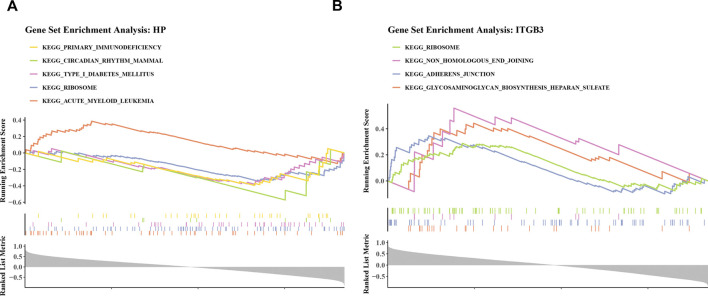
Gene Set Enrichment Analysis (GSEA) of biomarkers. **(A)** GSEA results for HP showing significantly enriched pathways (|NES| > 1, P < 0.05) in the GSE26168 dataset. Samples were divided into high and low HP expression groups based on median expression. The enrichment score (ES) curve (top panel of each plot) shows the running sum statistic as the analysis moves down the ranked gene list. The vertical black bars (middle panel, “hits”) indicate the positions of genes belonging to the tested gene set. The bottom panel shows the ranked list metric (Signal2Noise). Seven pathways were significantly enriched, with representative pathways shown including ribosome, primary immunodeficiency, and type I diabetes mellitus. NES, normalized enrichment score. **(B)** GSEA results for ITGB3 showing significantly enriched pathways (|NES| > 1, P < 0.05). Four pathways were significantly enriched, including glycosaminoglycan biosynthesis - heparan sulfate, adherens junction, and ribosome. SELP showed no significant enrichment under the same criteria.

Given the limited sample size of the training dataset (n = 17), the following GSEA results should be interpreted as exploratory. The GSEA observed that pathways associated with the expression of HP and ITGB3 were 7 and 4, respectively (Supplementary Tables 7, 8). Interestingly, SELP was not significantly enriched in pathways under the thresholds of |NES| > 1 and p < 0.05. Specifically, HP was remarkably enriched in the pathways of ribosome, primary immunodeficiency, and type I diabetes mellitus, while ITGB3 was enriched in glycosaminoglycan biosynthesis heparan sulfate, adherens junction, and ribosome (Figures 3A,B). GSEA in the GSE18309 dataset showed that HP was enriched in proteasome, ubiquitin-mediated proteolysis, neuroactive ligand–receptor interaction, and ECM-receptor interaction pathways, while ITGB3 was enriched in ECM-receptor interaction and neuroactive ligand-receptor interaction. Both T2DM and MCI shared enrichment in vascular and neural signaling pathways, but enrichment was weaker and less extensive in MCI, consistent with its earlier disease stage (Supplementary Figures 3A,B).

### A strong correlation was demonstrated between biomarkers and immune cells

**FIGURE 4:**
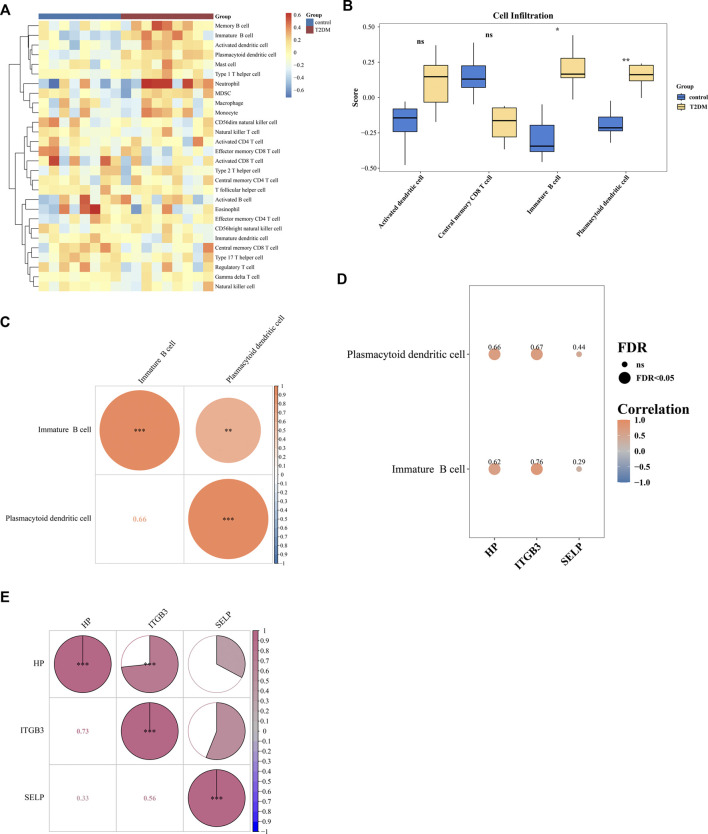
Correlation between biomarkers and immune cell infiltration in T2DM. **(A)** Heatmap showing the enrichment scores of 28 immune cell types in individual samples from the GSE26168 dataset, calculated using single-sample Gene Set Enrichment Analysis (ssGSEA). Each row represents an immune cell type, each column represents a sample. Colors indicate relative enrichment score, with red representing higher infiltration and blue representing lower infiltration. Hierarchical clustering was applied to both rows and columns. **(B)** Box plots comparing the infiltration levels of four significantly different immune cell types between T2DM patients and healthy controls in the GSE26168 dataset. P-values were calculated using the Wilcoxon rank-sum test with Benjamini-Hochberg FDR correction. *FDR <0.05, **FDR <0.01; ns, not significant. **(C)** Correlation matrix showing Spearman correlations among the two differentially expressed immune cell types in the GSE26168 dataset. Color intensity indicates correlation strength (red for positive correlation, blue for negative correlation). Numbers indicate correlation coefficients. Significance threshold: BH-adjusted p < 0.05 and |cor| > 0.3. **(D)** Dot plot showing Spearman correlations between the three biomarkers (HP, ITGB3, SELP) and the two FDR-significant immune cell types. Dot size indicates significance (filled: FDR <0.05; open: ns). Color intensity indicates correlation strength (red for positive, blue for negative). Numbers indicate Spearman correlation coefficients. **(E)** Correlation matrix showing Spearman correlations among the three biomarkers (HP, ITGB3, SELP) in the GSE26168 dataset.

Based on the samples in GSE26168, the relative composition of all cell types in each sample was shown in Figure 4A. The results showed that compared with the control group, Neutrophils constituted the highest proportion of cells in T2DM group, suggesting that the composition of immune cells in the immune microenvironment changed under the T2DM condition (p < 0.05). Following Benjamini-Hochberg FDR correction, 2 immune cell types exhibited significant differences between T2DM and control samples: immature B cells (d = 0.509, 95% CI: 0.263–0.738, FDR = 0.014) and plasmacytoid dendritic cells (d = 0.376, 95% CI: 0.186–0.457, FDR = 0.009) (Figure 4B). Correlation analysis revealed a significant positive correlation between immature B cells and plasmacytoid dendritic cells (cor = 0.66, FDR-adjusted p < 0.05) (Figure 4C). Additionally, Biomarker-immune cell correlation analysis demonstrated that HP showed significant positive correlations with plasmacytoid dendritic cells (cor = 0.67, 95%CI: 0.3291–0.8713, FDR = 0.0073) and immature B cells (cor = 0.62, 95%CI: 0.255–0.816, FDR = 0.0114); ITGB3 exhibited the strongest positive correlations with plasmacytoid dendritic cells (cor = 0.67, 95%CI: 0.353–0.819, FDR = 0.0073) and immature B cells (cor = 0.76, 95%CI: 0.432–0.902, FDR = 0.0021); SELP showed no significant correlation with either differential immune cell type (all FDR-adjusted p > 0.05) (Figure 4D).

Notably, biomarkers HP and ITGB3 showed a significant positive correlation (cor = 0.73, p < 0.05), while ITGB3 and SELP demonstrated a significant correlation (cor = 0.56, p < 0.05). However, no significant correlation was observed between HP and SELP (p > 0.05) (Figure 4E). The significant correlation between the biomarkers and the abundance of immune cells implied that HP, ITGB3, and SELP might have potential roles in regulating the immune microenvironment of T2DM.

The same immune infiltration analysis in the MCI dataset (GSE18309) showed a trend toward increased monocytes, macrophages, and neutrophils; however, no cell type remained significant after BH-FDR correction. Compared to T2DM, the weaker immune signal in MCI may reflect a less advanced stage of immune activation (Supplementary Figure 3C).

### Tissue and cellular localization of biomarkers

**FIGURE 5:**
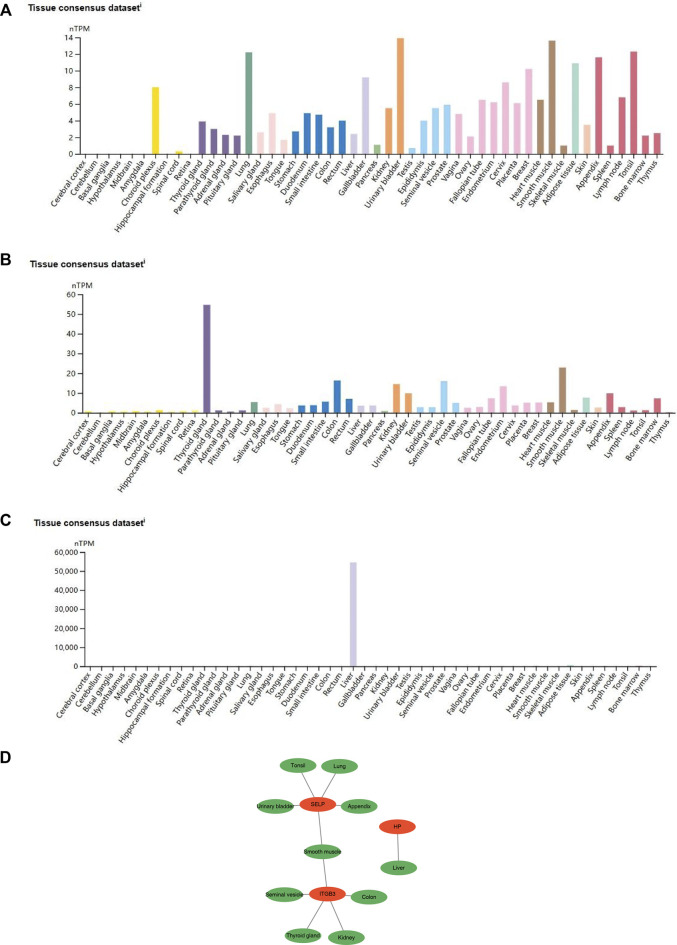
Tissue and cellular localization of biomarkers. **(A–C)** Tissue expression profiles of SELP **(A)**, ITGB3 **(B)**, and HP **(C)** obtained from the Human Proteome Atlas (HPA) database. Bar graphs show relative expression levels across different human tissues. SELP is primarily distributed in urinary bladder, lung, and appendix tonsil. ITGB3 is expressed in multiple tissues including thyroid gland, seminal vesicle, and kidney. HP shows extremely high tissue specificity, with expression predominantly in the liver. **(D)** Tissue-biomarker network diagram constructed using Cytoscape. Blue nodes represent tissues, orange nodes represent biomarkers. Edges connect biomarkers to tissues where they are expressed. Both SELP and ITGB3 are co-localized in smooth muscle tissue.

In the tissue-biomarker network, neither SELP nor ITGB3 exhibited tissue specificity, while HP demonstrated extremely high tissue specificity, being expressed exclusively in the liver (Figures 5A–C). Specifically, SELP was primarily distributed in the urinary bladder, lung, and appendix tonsil, whereas ITGB3 was expressed in tissues such as the thyroid gland, seminal vesicle, and kidney. Both SELP and ITGB3 were co-localized in smooth muscle tissue (Figure 5D).

Furthermore, subcellular localization results indicated that all biomarkers, HP, ITGB3, and SELP, were primarily localized to the extracellular (Confidence = 5). Besides, both SELP and ITGB3 are predominantly localized to the plasma membrane, consistent with their well-characterized roles as membrane-associated proteins. (Supplementary Table 9). Given the lack of UniProt support for GeneCards’ nuclear annotation of ITGB3, only its classical plasma membrane localization was considered to maintain functional and biological accuracy.

### *In silico* predicting candidate compounds targeting biomarkers

First, a total of 2,429 drug-biomarker interactions were predicted in the CMap database. Table 1 presents the top 10 and bottom 10 candidate compounds (by scores). For example, drugs such as calcifediol (vitamin D receptor agonist) and meclofenamic acid (cyclooxygenase inhibitor) exhibited high negative scores, indicating suggesting their gene expression signatures may computationally oppose the disease-associated transcriptomic pattern. TCM associations predicted by the Coremine Medical database are summarized in Supplementary Table 10 as exploratory text-mining outputs.

**TABLE 1:** Top 10 and bottom 10 potential compounds for biomarkers.

Score	ID	Name	Description
99.93	BRD-K22631935	Neurodazine	Neurogenesis of non-pluripotent C2C12 myoblast inducer
99.75	BRD-K03440695	Boldine	Acetylcholine receptor antagonist
99.72	BRD-K46212057	Voriconazole	Cytochrome P450 inhibitor
99.68	BRD-K04111260	Raclopride	Dopamine receptor antagonist
99.65	BRD-K02965346	SU-11274	Hepatocyte growth factor receptor inhibitor
99.65	BRD-K52397688	Amperozide	Dopamine receptor antagonist
99.58	BRD-K66896231	BRD-K66896231	Acetylcholinesterase inhibitor
99.58	BRD-K21283037	Riluzole	Glutamate inhibitor
99.58	BRD-K48168960	Propylthiouracil	Thyroid peroxidase inhibitor
99.47	BRD-K16551401	PNU-22394	Serotonin receptor agonist
−99.47	BRD-K05395900	Nicotine	Acetylcholine receptor agonist
−99.58	BRD-A47706533	L-BSO	Glutathione transferase inhibitor
−99.58	BRD-K60476892	YC-1	Guanylyl cyclase activator
−99.65	BRD-K04146668	GW-441756	Growth factor receptor inhibitor
−99.68	BRD-A03216249	Mepivacaine	Potassium channel blocker
−99.75	BRD-K05658747	Raltegravir	HIV integrase inhibitor
−99.75	BRD-A65615053	Zacopride	Serotonin receptor antagonist
−99.79	BRD-A65145453	ATPA	Glutamate receptor agonist
−99.89	BRD-K50398167	Meclofenamic-acid	Cyclooxygenase inhibitor
−99.93	BRD-K77175907	Calcifediol	Vitamin D receptor agonist

### Clinical experimental results

**FIGURE 6:**
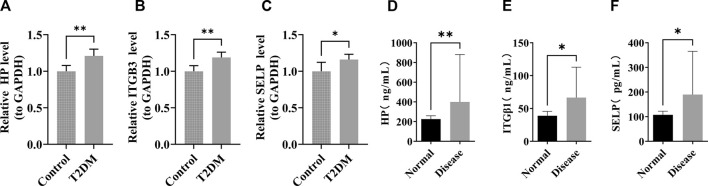
The mRNA and serum protein levels of biomarker expression in clinical samples. **(A–C)** mRNA expression levels of HP, ITGB3, and SELP in blood from T2DM patients (n = 5) and healthy controls (n = 5), as determined by RT-qPCR. Expression was normalized to GAPDH. Data are presented as mean ± SD. Comparisons between groups were performed using an independent samples t-test. **(D–F)** Serum concentrations of HP, ITGβ1, and SELP in T2DM patients (n = 11) and healthy controls (n = 10), as measured by ELISA. Data are presented as mean ± SD. Comparisons between groups were performed using an independent samples t-test. *P < 0.05; **P < 0.01.

RT-qPCR analysis indicated that, when juxtaposed with the control group, the expression levels of HP (P = 0.0050), ITGB3 (P = 0.0038), and SELP (P = 0.0358) were markedly increased in individuals diagnosed with T2DM (Figures 6A–C). These findings were corroborated at the protein level: serum concentrations of HP, ITGβ1, and SELP were similarly elevated in T2DM patients (n = 11) compared with healthy controls (n = 10), as determined by ELISA (all P < 0.05; Figures 6D–F). These observations aligned closely with the trends unearthed by our prior bioinformatics analyses, thereby providing additional validation for the relevance and reliability of these biomarkers in the pathophysiological context of T2DM. Effect sizes (Cohen’s d) for HP (2.46), ITGB3 (2.52), and SELP (1.56) indicated substantial biological impacts (Supplementary Table 11). Based on the smallest observed effect size (d ≈ 1.6), a requirement of n = 6 per group (80% power, α = 0.05). Our study utilized n = 5 per group, aligning closely with this calculated threshold for robust statistical inference.

## Discussion

T2DM is closely linked to MCI through insulin resistance, which in turn interacts bidirectionally with abnormal uric acid metabolism, forming a complex pathogenic network. The mechanisms underlying these interactions require further elucidation. Investigating biomarkers that bridge these conditions is crucial for early diagnosis, prognosis, and therapeutic development. This study integrated transcriptomic data from T2DM, MCI, and uric acid metabolism to identify candidate biomarkers co-dysregulated across these conditions. Functional analyses were mainly conducted in the T2DM dataset due to its larger sample size, while supplementary analyses in MCI showed partially overlapping pathway enrichment, supporting shared underlying mechanisms. Identification of biomarkers related to uric acid metabolism in T2DM and MCI, differentially expressed genes based on transcriptome data, combined with two machine learning methods. Analyze the functions, immune characteristics, and potential regulatory mechanisms of biomarkers through bioinformatics methods. We used two types of machine learning to screen for three feature genes (HP, ITGB3, and SELP). The expression levels of the three characteristic genes mentioned above were validated, and subsequent analysis was conducted on the obtained biomarkers. Although the uric acid-related gene set was initially derived from a keyword-based GeneCards search, which may include indirect associations. However, sensitivity analysis using a biologically constrained 585-gene set consistently identified HP, ITGB3, and SELP, supporting robustness to gene set definition. Column charts were constructed for the biomarkers HP, ITGB3, and SELP. In the independent validation cohort (GSE95849, n = 12), all three biomarkers achieved AUC >0.9, though the small sample size warrants cautious interpretation of these estimates.

The HP gene encodes Haptoglobin (Hp), a hemoglobin-binding protein synthesized primarily in the liver and brain that prevents iron loss and oxidative damage. GSEA analysis revealed HP co-enrichment in seven pathways, with the top five being acute myeloid leukemia, ribosome, type 1 diabetes mellitus, circadian rhythm (mammalian), and primary immunodeficiency. These enrichment patterns highlight HP’s multifaceted biological roles: its involvement in acute myeloid leukemia and primary immunodeficiency pathways indicates a central role in immune regulation, with HP protein types associating with certain acute myeloid leukemias [38] and showing therapeutic potential for cerebral ischemia through regulation of myeloid immune cells in T2DM’s chronic inflammatory state [39]. The type 1 diabetes mellitus pathway enrichment may reflect shared immune dysregulation signatures between T1DM and T2DM, as both involve inflammatory and immune-mediated processes affecting pancreatic function [40]. However, this co-enrichment should not be interpreted as evidence of identical pathological mechanisms, given that T1DM and T2DM are pathophysiologically distinct diseases; this finding warrants cautious interpretation and further mechanistic investigation. Ribosome pathway enrichment indicates extensive involvement in protein synthesis, reflecting metabolic reprogramming under T2DM pathological conditions related to compensatory insulin secretion or inflammatory factor production [41], while circadian rhythm pathway enrichment suggests that genes co-expressed with HP may be involved in clock-regulated biological processes [27]; however, whether this reflects a direct mechanistic link to glucose metabolism or insulin secretion rhythms in T2DM remains speculative and requires dedicated experimental validation. HP gene polymorphisms (Hp1-1, Hp2-1, Hp2-2) critically affect cognitive function through oxidative stress and inflammatory regulation, with the Hp1-1 genotype associating with increased risk of cerebral small vessel diseases, including lacunar infarction [42] and white matter lesions [43]. T2DM patients with poor glycemic control and Hp1-1 genotype face higher cognitive impairment risk, particularly in attention and working memory [44], through mechanisms including cerebrovascular disorders [45], hippocampal volume reduction [46], and increased depression susceptibility related to white matter damage and frontal lobe atrophy [47]. Notably, the observed HP upregulation reflects overall transcriptomic expression rather than genotype-specific effects, and whether it preferentially corresponds to a specific Hp phenotype remains unclear. Our PCR results demonstrated significant HP gene upregulation in T2DM patients compared to controls, consistent with a potential pathological role; however, its specific function in T2DM-associated MCI requires further experimental investigation. Notably, HP achieved an AUC of 1.000 in the validation dataset; however, this may reflect overestimation due to the small sample size (n = 6 per group). Despite using an independent cohort, the risk of optimistic bias remains, and further validation in larger datasets is required.

The ITGB3 gene encodes integrin β3, a cell surface protein that forms heterodimeric integrins with alpha chains to mediate cell adhesion to extracellular matrix and mechanical force signal transduction. GSEA analysis revealed ITGB3 enrichment in four pathways: glycosaminoglycan biosynthesis heparan sulfate chondroitin sulfate, adherens junction, non-homologous end joining, and ribosome, depicting its multifaceted roles in T2DM pathology. The enrichment in glycosaminoglycan biosynthesis and adherens junction pathways directly reflects ITGB3’s core function in maintaining vascular integrity, as glycosaminoglycans are critical basement membrane components essential for vascular wall stability and signal transduction, suggesting ITGB3’s pivotal role in diabetic vascular complications including atherosclerosis and nephropathy [48]. The adherens junction enrichment emphasizes its role in preserving endothelial barrier function and intercellular communication, whose disruption represents an early event in vascular leakage and dysfunction. The unexpected non-homologous end joining pathway enrichment indicates genes correlated with ITGB3 participate in DNA damage repair, reflecting substantial genotoxic stress under diabetic high glucose and oxidative conditions, while ribosome pathway enrichment confirms protein synthesis alterations in disease states. ITGB3’s complex role in diabetic vascular pathology manifests through contrasting mechanisms: integrin β3 glycoprotein functions critically in platelet physiology [49], with ITGB3 variations causing Glanzmann thrombasthenia [50], and ITGB3 deficiency promoting atherosclerosis in hyperlipidemic mice [51]. Mechanistically, miR-351 inhibits the ITGB3/PIK3R1/Akt pathway to promote diabetic atherosclerosis and induce endothelial damage with lipid accumulation, while silencing miR-351 upregulates ITGB3 to activate the PIK3R1/Akt pathway, producing anti-apoptotic and endothelial protective effects [48]. Conversely, oscillatory shear stress upregulates ITGB3 expression, impairing autophagosome-lysosome fusion and increasing endothelial senescence markers (P16, P21), exacerbating endothelial dysfunction [52]. These diabetic vascular pathologies constitute key mechanisms underlying cognitive impairment, as cerebrovascular endothelial damage increases blood-brain barrier permeability, reduces cerebral perfusion, and triggers neuroinflammation [53]. These observations suggest that ITGB3 may be involved in the pathological continuum connecting T2DM vascular complications and cognitive decline; however, direct evidence linking ITGB3 dysregulation to cognitive outcomes in T2DM patients remains to be established.

The SELP gene encodes P-selectin, a key molecule in platelet activation and inflammatory responses, which mediates the interactions between platelets, endothelial cells, and leukocytes. SELP was not significantly enriched in KEGG pathways, which may imply that its mechanism of action is more specific and not reflected at the broad pathway level, but rather through more refined protein interactions. Its expression changes primarily affect the cell membrane surface and platelet function, processes that may not be fully captured in traditional metabolic pathway databases.

In this study, RT-qPCR detection revealed a significant increase in SELP expression in patients with type 2 diabetes, suggesting that P-selectin may be involved in the pathological process of diabetes. The role of P-selectin in diabetes associated cognitive impairment is not yet fully understood, with existing research suggesting it may have a dual role. Animal studies have shown that P-selectin encoded by the human SELP gene can promote the formation of atherosclerosis in apolipoprotein E-deficient mice [54], indicating that excessive activation of P-selectin may contribute to the atherosclerotic process by promoting vascular inflammation. Patients with diabetes often exhibit platelet overactivation and a chronic inflammatory state [55], and the leukocyte-platelet-endothelial cell interactions mediated by P-selectin may exacerbate microvascular dysfunction, thereby affecting cerebrovascular health. However, a prospective cohort study [37] found that the minor allele of the SELP gene 1087G/A single-nucleotide polymorphism (SNP) was associated with reduced cognitive impairment, suggesting that this gene variant may exert a protective effect by modulating the expression or function of P-selectin. This implies that a moderate reduction or functional alteration of P-selectin might be beneficial for maintaining cognitive function. In Alzheimer’s disease (AD) research [56], studies found that P-selectin levels were decreased in AD patients, with the most significant decrease observed in patients with the most severe cognitive decline, accompanied by elevated levels of inflammatory factors such as IL-8, IFN-γ, MCP-1, and VEGF. Overall, SELP shows stage-dependent dynamics: elevated in early T2DM but reduced in advanced AD; cognitive impact likely depends on functional regulation rather than absolute levels, warranting longitudinal studies.

Combined with the significant upregulation of SELP in type 2 diabetes patients in this study and the literature evidence, it is suggested that P-selectin may exert context-dependent effects in diabetes-related cognitive dysfunction, though this hypothesis requires dedicated experimental testing. In this study, multiple drugs were predicted in the CMap database, including neurodazine, boldine, etc. CMap database predictions indicate that Boldine and BRD-K66896231 (an acetylcholinesterase inhibitor), acting as acetylcholine receptor antagonists/inhibitors, may hypothetically modulate the cholinergic anti-inflammatory pathway, suggesting a potential direction for future investigation into chronic inflammation in T2DM. However, these inferences are based solely on computational predictions from cancer cell line data and require experimental validation.

Serotonin is a key neurotransmitter that regulates the sleep-wake cycle and emotions, and closely interacts with the biological clock. PNU-22394, as a serotonin receptor agonist, can target the serotonin system and represents a computationally nominated candidate for correcting HP-related circadian rhythm disorders and improving glucose metabolism.

Calcifediol (a vitamin D receptor agonist) was computationally predicted to oppose biomarker expression patterns based on CMap scoring. Studies have shown that vitamin D receptors significantly reduce fasting blood glucose, serum insulin levels, and improve insulin resistance in rats [57]. Insulin resistance is a common pathological basis for both T2DM and cognitive impairment. In terms of neuroprotection, vitamin D can downregulate the α1C/α1D subunits of L-type voltage-gated calcium channels (L-VGCCs) in hippocampal neurons, reducing excitotoxic injury caused by calcium overload, particularly under conditions of brain aging or ischemia. Furthermore, serum vitamin D levels are positively correlated with brain volume, and deficiency may lead to brain atrophy, increasing the risk of cognitive decline [58]. Given the widespread vitamin D deficiency in diabetic patients [59], vitamin D supplementation may warrant investigation as a potential adjunct strategy in T2DM-related cognitive decline, and further clinical validation is required before any therapeutic inference can be drawn.

The multi-component, multi-target nature of traditional Chinese medicine is derived entirely from database-driven text-mining and *in silico* analyses without experimental validation, and should therefore be interpreted with caution. Thus, caution is required when interpreting herbal materials, particularly those from animal or conservation-sensitive sources. Future studies should prioritize ethically acceptable and sustainable candidates. Nevertheless, systematic elucidation of their mechanisms and clinical application value still requires further validation through methods such as network pharmacology predictions, *in vitro* cell experiments, and animal model studies.

This study systematically identified biomarkers related to MCI and uric acid metabolism in T2DM through databases and various bioinformatics methods, including HP, ITGB3, and SELP. Through analysis, it was found that biomarkers were significantly enriched in pathways related to ribosomes, immune deficiencies, and circadian rhythms, further revealing the potential function of biomarkers in T2DM. Subsequently, therapeutic compounds and traditional Chinese medicines related to biomarkers were predicted through candidate compounds and herbal medicines, expanding the direction of targeted therapy for T2DM. Finally, RT qPCR was used to further validate the correlation and reliability of biomarkers in the pathology of T2DM. Although this study has obtained relatively systematic results, there are still certain limitations. Firstly, the main mechanism research relies on bioinformatics predictions and lacks cell or animal experiments to verify the specific regulatory functions of biomarkers in T2DM (such as gene knockout/overexpression experiments); Secondly, the limited sample sizes across all cohorts (MCI discovery: n = 3; RT-qPCR validation: n = 5 per group; external validation: n = 12) constrain statistical power and generalizability. Although large effect sizes (Cohen’s d: 1.56–2.52) and LOOCV-confirmed feature stability partially mitigate this concern, the perfect AUC (1.000) for HP likely reflects a sample size artifact. These findings should therefore be considered hypothesis-generating, pending validation in larger independent cohorts (n ≥ 50 per group). Furthermore, the complexity and diversity of biological systems may not fully reflect actual biological processes, and the results need to be validated through experiments to confirm their authenticity and reliability. The tri-disease framework is mainly supported by gene-level overlap, with downstream analyses largely limited to T2DM datasets. Although MCI analyses provided preliminary support, the small sample size (n = 3 per group) and lack of MCI-specific validation limit conclusions about MCI-specific mechanisms.

In the future, it is necessary to deepen the exploration of mechanisms through multi-organizational sample analysis and functional experiments.

## Conclusion

We identified HP, ITGB3, and SELP as biomarkers potentially associated with T2DM, MCI, and uric acid metabolism. These genes are involved in inflammatory, vascular, and metabolic pathways and correlate with immune cell infiltration. Our findings provide preliminary insights into shared molecular features across these conditions and suggest a potential link between metabolic dysregulation and cognitive impairment in T2DM. However, given the limited sample size and the exploratory nature of this study, the results should be considered hypothesis-generating.

## Data Availability

The datasets presented in this study can be found in online repositories. The names of the repository/repositories and accession number(s) can be found in the article/Supplementary Material.

## Footnotes

1
https://www.ncbi.nlm.nih.gov/geo/

2
https://www.genecards.org/

3
https://string-db.org/

4
https://genemania.org/

5
https://www.gsea-msigdb.org/gsea/msigdb/

6
https://www.proteinatlas.org/

7
https://clue.io/

8
http://www.coremine.com

9
https://www.graphpad.com/

## References

[1] Magliano DJ Sacre JW Harding JL Gregg EW Zimmet PZ Shaw JE . Young-onset type 2 diabetes mellitus - implications for morbidity and mortality. Nat Rev Endocrinol (2020) 16:321–31. 10.1038/s41574-020-0334-z 32203408

[2] Yan Y Wu T Zhang M Li C Liu Q Li F . Prevalence, awareness and control of type 2 diabetes mellitus and risk factors in Chinese elderly population. BMC Public Health (2022) 22:1382. 10.1186/s12889-022-13759-9 35854279 PMC9295461

[3] Mirzadeh Z Faber CL Schwartz MW . Central nervous system control of glucose homeostasis: a therapeutic target for type 2 diabetes? Annu Rev Pharmacol Toxicol (2022) 62:55–84. 10.1146/annurev-pharmtox-052220-010446 34990204 PMC8900291

[4] You Y Liu Z Chen Y Xu Y Qin J Guo S The prevalence of mild cognitive impairment in type 2 diabetes mellitus patients: a systematic review and meta-analysis. Acta Diabetol (2021) 58:671–85. 10.1007/s00592-020-01648-9 33417039

[5] Srikanth V Sinclair AJ Hill-Briggs F Moran C Biessels GJ . Type 2 diabetes and cognitive dysfunction-towards effective management of both comorbidities. Lancet Diabetes Endocrinol (2020) 8:535–45. 10.1016/s2213-8587(20)30118-2 32445740

[6] Ehtewish H Arredouani A El-Agnaf O . Diagnostic, prognostic, and mechanistic biomarkers of diabetes mellitus-associated cognitive decline. Int J Mol Sci (2022) 23:6144. 10.3390/ijms23116144 35682821 PMC9181591

[7] Bai W Chen P Cai H Zhang Q Su Z Cheung T Worldwide prevalence of mild cognitive impairment among community dwellers aged 50 years and older: a meta-analysis and systematic review of epidemiology studies. Age Ageing (2022) 51. 10.1093/ageing/afac173 35977150

[8] Sędzikowska A Szablewski L . Insulin and insulin resistance in Alzheimer's Disease. Int J Mol Sci (2021) 22:22. 10.3390/ijms22189987 34576151 PMC8472298

[9] Asma Sakalli A Küçükerdem HS Aygün O . What is the relationship between serum uric acid level and insulin resistance? A case-control study. Medicine (Baltimore) (2023) 102:e36732. 10.1097/md.0000000000036732 38206747 PMC10754590

[10] Johnson RJ Nakagawa T Sanchez-Lozada LG Shafiu M Sundaram S Le M Sugar, uric acid, and the etiology of diabetes and obesity. Diabetes (2013) 62:3307–15. 10.2337/db12-1814 24065788 PMC3781481

[11] Kuwabara M Kanbay M Hisatome I . Tips and pitfalls in uric acid clinical research. Hypertens Res (2023) 46:771–3. 10.1038/s41440-022-01148-z 36577846

[12] Yanai H Adachi H Hakoshima M Katsuyama H . Molecular biological and clinical understanding of the pathophysiology and treatments of hyperuricemia and its association with metabolic syndrome, cardiovascular diseases and chronic kidney disease. Int J Mol Sci (2021) 22:22. 10.3390/ijms22179221 34502127 PMC8431537

[13] Gherghina ME Peride I Tiglis M Neagu TP Niculae A Checherita IA . Uric acid and oxidative stress-relationship with cardiovascular, metabolic, and renal impairment. Int J Mol Sci (2022) 23:23. 10.3390/ijms23063188 35328614 PMC8949471

[14] Liu YR Wang JQ Li J . Role of NLRP3 in the pathogenesis and treatment of gout arthritis. Front Immunol (2023) 14:1137822. 10.3389/fimmu.2023.1137822 37051231 PMC10083392

[15] Chen C Li X Lv Y Yin Z Zhao F Liu Y High blood uric acid is associated with reduced risks of mild cognitive impairment among older adults in China: a 9-Year prospective cohort study. Front Aging Neurosci (2021) 13:747686. 10.3389/fnagi.2021.747686 34720995 PMC8552040

[16] Lv H Sun J Zhang T Hui Y Li J Zhao X Associations of serum uric acid variability with neuroimaging metrics and cognitive decline: a population-based cohort study. BMC Med (2024) 22:256. 10.1186/s12916-024-03479-9 38902722 PMC11188528

[17] Karolina DS Armugam A Tavintharan S Wong MTK Lim SC Sum CF MicroRNA 144 impairs insulin signaling by inhibiting the expression of insulin receptor substrate 1 in type 2 diabetes mellitus. PLoS One (2011) 6(8):e22839. 10.1371/journal.pone.0022839 21829658 PMC3148231

[18] Ye C Fu Y Zhou X Zhou F Zhu X Chen Y . Identification and validation of NAD+ metabolism-related biomarkers in patients with diabetic peripheral neuropathy. Front Endocrinol (Lausanne) (2024) 15:1309917. 10.3389/fendo.2024.1309917 38464965 PMC10920259

[19] Zhang Y Deng S Zhong H Liu M Ding J Geng R Exploration and clinical verification of the blood Co-Expression genes of type 2 diabetes mellitus and mild cognitive dysfunction in the elderly. Biomedicines (2023) 11:11. 10.3390/biomedicines11040993 37189611 PMC10135937

[20] Stelzer G Rosen N Plaschkes I Zimmerman S Twik M Fishilevich S The GeneCards suite: from gene data mining to disease genome sequence analyses. Curr Protoc Bioinforma (2016) 54(1):1.30.1–.30.33. 10.1002/cpbi.5 27322403

[21] Ritchie ME Phipson B Wu D Hu Y Law CW Shi W Limma powers differential expression analyses for RNA-sequencing and microarray studies. Nucleic Acids Res (2015) 43:e47. 10.1093/nar/gkv007 25605792 PMC4402510

[22] Gustavsson EK Zhang D Reynolds RH Garcia-Ruiz S Ryten M . Ggtranscript: an R package for the visualization and interpretation of transcript isoforms using ggplot2. Bioinformatics (2022) 38:3844–6. 10.1093/bioinformatics/btac409 35751589 PMC9344834

[23] Gu Z . Complex heatmap visualization. Imeta (2022) 1:e43. 10.1002/imt2.43 38868715 PMC10989952

[24] Gu Z Gu L Eils R Schlesner M Brors B . Circlize implements and enhances circular visualization in R. Bioinformatics (2014) 30:2811–2. 10.1093/bioinformatics/btu393 24930139

[25] Chen H Boutros PC . VennDiagram: a package for the generation of highly-customizable Venn and Euler diagrams in R. BMC Bioinformatics (2011) 12:35. 10.1186/1471-2105-12-35 21269502 PMC3041657

[26] Wu T Hu E Xu S Chen M Guo P Dai Z clusterProfiler 4.0: a universal enrichment tool for interpreting omics data. Innovation (Camb) (2021) 2:100141. 10.1016/j.xinn.2021.100141 34557778 PMC8454663

[27] Amaral FG Castrucci AM Cipolla-Neto J Poletini MO Mendez N Richter HG Environmental control of biological rhythms: effects on development, fertility and metabolism. J Neuroendocrinol (2014) 26:603–12. 10.1111/jne.12144 24617798

[28] Su G Morris JH Demchak B Bader GD . Biological network exploration with cytoscape 3. Curr Protoc Bioinformatics (2014) 47(8.13):11–24. 10.1002/0471250953.bi0813s47 25199793 PMC4174321

[29] Li Y Lu F Yin Y . Applying logistic LASSO regression for the diagnosis of atypical Crohn's disease. Sci Rep (2022) 12:11340. 10.1038/s41598-022-15609-5 35790774 PMC9256608

[30] Hou N Li M He L Xie B Wang L Zhang R Predicting 30-days mortality for MIMIC-III patients with sepsis-3: a machine learning approach using XGboost. J Translational Medicine (2020) 18:462. 10.1186/s12967-020-02620-5 33287854 PMC7720497

[31] Gao CH Yu G Cai P . ggVennDiagram: an intuitive, Easy-to-Use, and highly customizable R package to generate Venn diagram. Front Genet (2021) 12:706907. 10.3389/fgene.2021.706907 34557218 PMC8452859

[32] Robin X Turck N Hainard A Tiberti N Lisacek F Sanchez JC pROC: an open-source package for R and S+ to analyze and compare ROC curves. BMC Bioinformatics (2011) 12:77. 10.1186/1471-2105-12-77 21414208 PMC3068975

[33] Zheng J Zhang T Guo W Zhou C Cui X Gao L Integrative analysis of multi-omics identified the prognostic biomarkers in acute myelogenous leukemia. Front Oncol (2020) 10:591937. 10.3389/fonc.2020.591937 33363022 PMC7758482

[34] Hänzelmann S Castelo R Guinney J . GSVA: gene set variation analysis for microarray and RNA-seq data. BMC Bioinformatics (2013) 14:7. 10.1186/1471-2105-14-7 23323831 PMC3618321

[35] Zhang X Chao P Zhang L Xu L Cui X Wang S Single-cell RNA and transcriptome sequencing profiles identify immune-associated key genes in the development of diabetic kidney disease. Front Immunol (2023) 14:1030198. 10.3389/fimmu.2023.1030198 37063851 PMC10091903

[36] Livak KJ Schmittgen TD . Analysis of relative gene expression data using real-time quantitative PCR and the 2(-Delta Delta C(T)) method. Methods (2001) 25:402–8. 10.1006/meth.2001.1262 11846609

[37] Mathew JP Podgoreanu MV Grocott HP White WD Morris RW Stafford-Smith M Genetic variants in P-selectin and C-reactive protein influence susceptibility to cognitive decline after cardiac surgery. J Am Coll Cardiol (2007) 49:1934–42. 10.1016/j.jacc.2007.01.080 17498578

[38] Nevo S Tatarsky I . Serum haptoglobin types and leukemia. Hum Genet (1986) 73:240–4. 10.1007/bf00401236 3460960

[39] Morimoto M Nakano T Egashira S Irie K Matsuyama K Wada M Haptoglobin regulates Macrophage/microglia-induced inflammation and prevents ischemic brain damage *via* binding to HMGB1. J Am Heart Assoc (2022) 11:e024424. 10.1161/jaha.121.024424 35243897 PMC9075294

[40] Wan BN Zhou SG Wang M Zhang X Ji G . Progress on haptoglobin and metabolic diseases. World J Diabetes (2021) 12:206–14. 10.4239/wjd.v12.i3.206 33758643 PMC7958475

[41] Murugesan S Yousif G Djekidel MN Gentilcore G Grivel JC Al Khodor S . Microbial and proteomic signatures of type 2 diabetes in an Arab population. J Transl Med (2024) 22:1132. 10.1186/s12967-024-05928-8 39707404 PMC11662572

[42] Costacou T Secrest AM Ferrell RE Orchard TJ . Haptoglobin genotype and cerebrovascular disease incidence in type 1 diabetes. Diab Vasc Dis Res (2014) 11:335–42. 10.1177/1479164114539713 24994788 PMC4134420

[43] Costacou T Rosano C Aizenstein H Mettenburg JM Nunley K Ferrell RE The haptoglobin 1 allele correlates with white matter hyperintensities in middle-aged adults with type 1 diabetes. Diabetes (2015) 64:654–9. 10.2337/db14-0723 25213335 PMC4303969

[44] Guerrero-Berroa E Ravona-Springer R Heymann A Schmeidler J Levy A Leroith D Haptoglobin genotype modulates the relationships of glycaemic control with cognitive function in elderly individuals with type 2 diabetes. Diabetologia (2015) 58:736–44. 10.1007/s00125-014-3487-2 25628235 PMC4352385

[45] Ravona-Springer R Heymann A Schmeidler J Guerrero-Berroa E Sano M Preiss R Haptoglobin 1-1 genotype is associated with poorer cognitive functioning in the elderly with type 2 diabetes. Diabetes Care (2013) 36:3139–45. 10.2337/dc12-2250 23990521 PMC3781506

[46] Livny A Ravona-Springer R Heymann A Priess R Kushnir T Tsarfaty G Haptoglobin 1-1 genotype modulates the association of glycemic control with hippocampal volume in elderly individuals with type 2 diabetes. Diabetes (2017) 66:2927–32. 10.2337/db16-0987 28860127 PMC5652603

[47] Livny A Schnaider Beeri M Heymann A Schmeidler J Moshier E Tzukran R The association of depressive symptoms with brain volume is stronger among diabetic elderly carriers of the haptoglobin 1-1 genotype compared to non-carriers. Front Endocrinol (Lausanne) (2019) 10:68. 10.3389/fendo.2019.00068 30809196 PMC6379325

[48] Li H Song D Liu Q Li L Sun X Guo J miR-351 promotes atherosclerosis in diabetes by inhibiting the ITGB3/PIK3R1/Akt pathway and induces endothelial cell injury and lipid accumulation. Mol Med (2022) 28:120. 10.1186/s10020-022-00547-9 36180828 PMC9523959

[49] Dahiya N Atreya C . MiRNA-103b downregulates ITGB3 and mediates apoptosis in *Ex Vivo* stored human platelets. Microrna (2021) 10:123–9. 10.2174/2211536610666210604121854 34086556

[50] Wang Z Xu Y Sun Y Wang S Dong M . Novel homozygous silent mutation of ITGB3 gene caused Glanzmann thrombasthenia. Front Pediatr (2022) 10:1062900. 10.3389/fped.2022.1062900 36704147 PMC9871544

[51] Weng S Zemany L Standley KN Novack DV La Regina M Bernal-Mizrachi C Beta3 integrin deficiency promotes atherosclerosis and pulmonary inflammation in high-fat-fed, hyperlipidemic mice. Proc Natl Acad Sci U S A (2003) 100:6730–5. 10.1073/pnas.1137612100 12746502 PMC164515

[52] Li S Jiang X Huang W Meng Q Pu L Sun B Oscillatory shear stress activates integrin β3, blocking autophagic flux in endothelial cells and promoting endothelial cells senescence. Biochim Biophys Acta Mol Cell Res (2025) 1872:119991. 10.1016/j.bbamcr.2025.119991 40412535

[53] Gorelick PB Scuteri A Black SE Decarli C Greenberg SM Iadecola C Vascular contributions to cognitive impairment and dementia: a statement for healthcare professionals from the american heart association/american stroke association. Stroke (2011) 42:2672–713. 10.1161/STR.0b013e3182299496 21778438 PMC3778669

[54] Zhang N Liu Z Yao L Mehta-D'souza P McEver RP . P-Selectin expressed by a human SELP transgene is atherogenic in apolipoprotein E-Deficient mice. Arterioscler Thromb Vasc Biol (2016) 36:1114–21. 10.1161/atvbaha.116.307437 27102967 PMC4882243

[55] R S Saharia GK Patra S Bandyopadhyay D Patro BK . Flow cytometry based platelet activation markers and state of inflammation among subjects with type 2 diabetes with and without depression. Sci Rep (2022) 12:10039. 10.1038/s41598-022-13037-z 35710773 PMC9203543

[56] Corsi MM Licastro F Porcellini E Dogliotti G Galliera E Lamont JL Reduced plasma levels of P-selectin and L-selectin in a pilot study from Alzheimer disease: relationship with neuro-degeneration. Biogerontology (2011) 12:451–4. 10.1007/s10522-011-9335-6 21484243

[57] Abdel-Rehim WM El-Tahan RA El-Tarawy MA Shehata RR Kamel MA . The possible antidiabetic effects of vitamin D receptors agonist in rat model of type 2 diabetes. Mol Cell Biochem (2019) 450:105–12. 10.1007/s11010-018-3377-x 29909574

[58] Liu Y Zhong Z Xie J Ni B Wu Y . Neuroprotective roles of vitamin D: bridging the gap between mechanisms and clinical applications in cognitive decline. Int J Mol Sci (2025) 26:26. 10.3390/ijms26157146 40806275 PMC12346440

[59] Berridge MJ . Vitamin D deficiency and diabetes. Biochem J (2017) 474:1321–32. 10.1042/bcj20170042 28341729

