# Supplementary figures and images for: Transcriptomics-based identification of shared biomarkers across type 2 diabetes, mild cognitive impairment, and uric acid metabolism
Source: Exp Biol Med (Maywood). 2026 Jul 6;251:11060. doi: 10.3389/ebm.2026.11060 (PMC13381339; doi:10.3389/ebm.2026.11060)

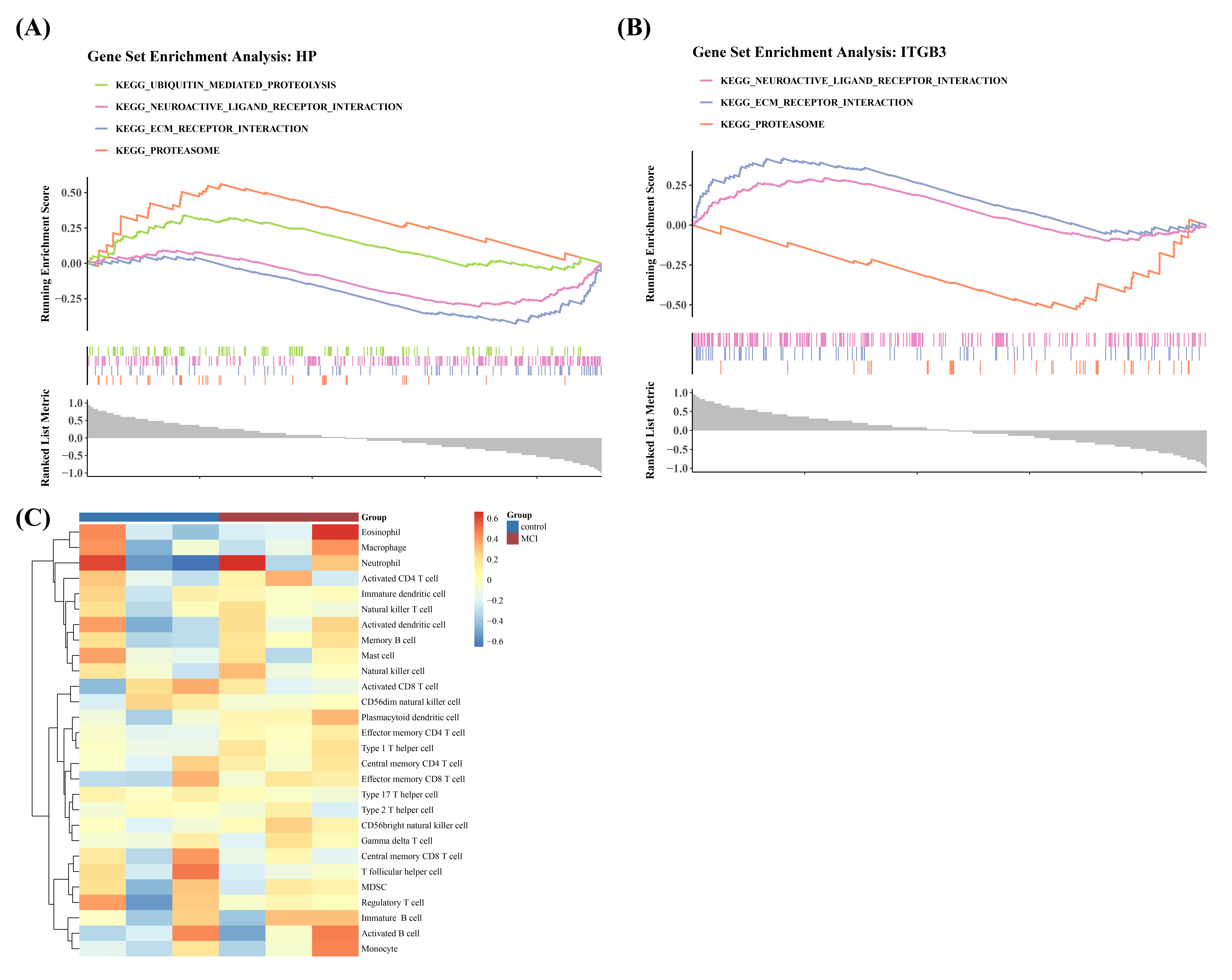

Supplement: Supplementary file 4 [file Image3.TIF]

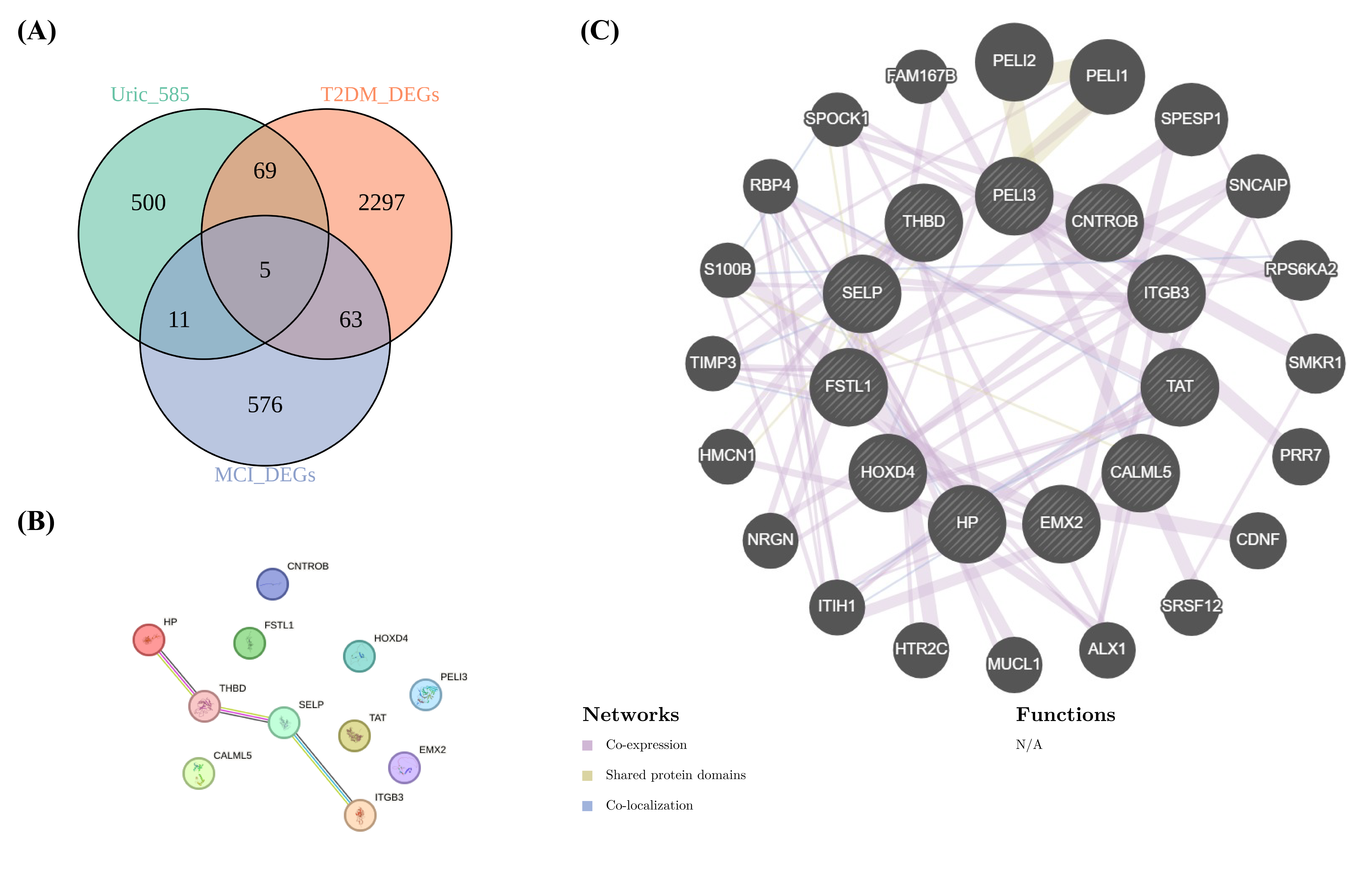

Supplement: Supplementary file 5 [file Image2.TIF]

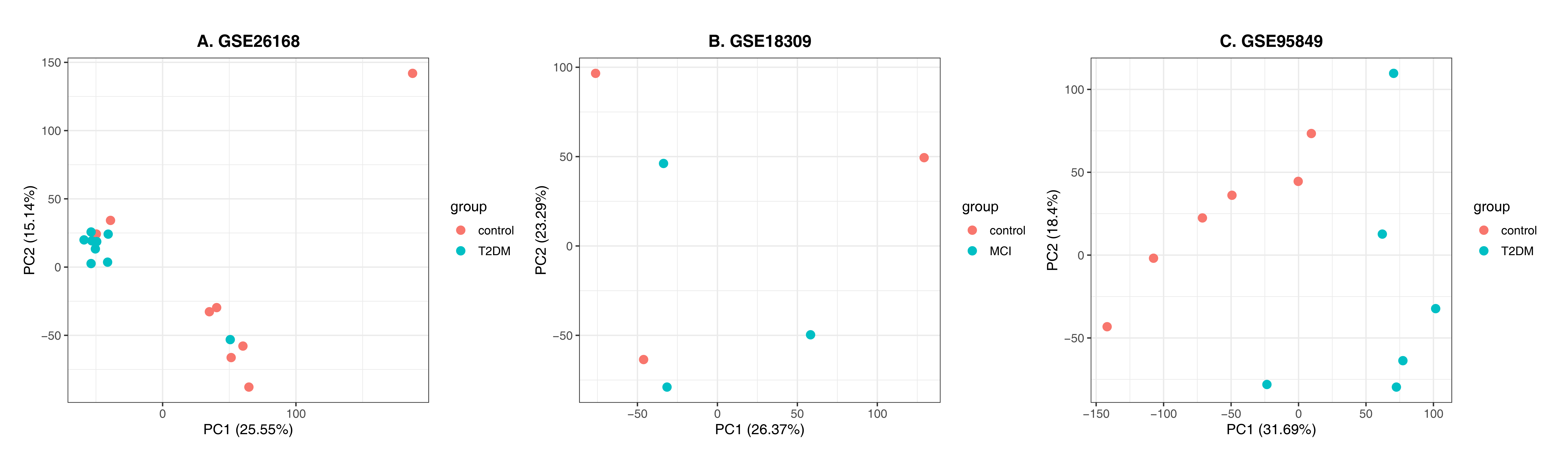

Supplement: Supplementary file 6 [file Image1.TIF]
